# Evolutionary dynamics of paroxysmal nocturnal hemoglobinuria

**DOI:** 10.1371/journal.pcbi.1006133

**Published:** 2018-06-18

**Authors:** Nathaniel Mon Père, Tom Lenaerts, Jorge M. Pacheco, David Dingli

**Affiliations:** 1 Interuniversity Institute of Bioinformatics in Brussels, ULB-VUB, Brussels, Belgium; 2 MLG, Département d’Informatique, Université Libre de Bruxelles, Brussels, Belgium; 3 AI lab, Computer Science Department, Vrije Universiteit Brussel, Brussels, Belgium; 4 Centro de Biologia Molecular e Ambiental, Universidade do Minho, Braga, Portugal; 5 Departamento de Matemática e Aplicações, Universidade do Minho, Braga, Portugal; 6 ATP-group, Porto Salvo, Portugal; 7 Division of Hematology and Department of Molecular Medicine, Mayo Clinic, Rochester, MN, United States of America; University of Minnesota, UNITED STATES

## Abstract

Paroxysmal nocturnal hemoglobinuria (PNH) is an acquired clonal blood disorder characterized by hemolysis and a high risk of thrombosis, that is due to a deficiency in several cell surface proteins that prevent complement activation. Its origin has been traced to a somatic mutation in the PIG-A gene within hematopoietic stem cells (HSC). However, to date the question of how this mutant clone expands in size to contribute significantly to hematopoiesis remains under debate. One hypothesis posits the existence of a selective advantage of PIG-A mutated cells due to an immune mediated attack on normal HSC, but the evidence supporting this hypothesis is inconclusive. An alternative (and simpler) explanation attributes clonal expansion to neutral drift, in which case selection neither favours nor inhibits expansion of PIG-A mutated HSC. Here we examine the implications of the neutral drift model by numerically evolving a Markov chain for the probabilities of all possible outcomes, and investigate the possible occurrence and evolution, within this framework, of multiple independently arising clones within the HSC pool. Predictions of the model agree well with the known incidence of the disease and average age at diagnosis. Notwithstanding the slight difference in clonal expansion rates between our results and those reported in the literature, our model results lead to a relative stability of clone size when averaging multiple cases, in accord with what has been observed in human trials. The probability of a patient harbouring a second clone in the HSC pool was found to be extremely low (${\sim 10}^{-8}$). Thus our results suggest that in clinical cases of PNH where two independent clones of mutant cells are observed, only one of those is likely to have originated in the HSC pool.

## Author summary

The mechanisms leading to expansion of HSC with mutations in the PIG-A gene that leads to the PNH phenotype remains unclear. Data so far suggests there is no intrinsic fitness advantage of the mutant cells compared to normal cells. Assuming neutral drift within the HSC compartment, we determined from first principles the incidence of the disease in a population, the average clone size in patients, the probability of clonal extinction, the likelihood of several separate clones coexisting in the HSC pool, and the expected expansion rate of a mutant clone. Our results are similar to what is observed in clinical practice. We also find that in such a model the probability of multiple PNH clones arising independently in the HSC pool is exceptionally small. This suggests that in clinical cases where more than one distinct clone is observed, all but one of the clones are likely to have emerged in cells that are downstream of the HSC population. We propose that PNH is perhaps the first disease where neutral drift alone may be responsible for clonal expansion leading to a clinical problem.

## Introduction

Paroxysmal nocturnal hemoglobinuria (PNH) is an acquired disorder of hematopoietic stem cells (HSC) due to a somatic mutation in the PIG-A gene [1, 2]. Loss of function or hypofunction mutations in this gene result in loss of or reduced ability to synthesize the glycosylphosphatidylinositol (GPI) anchor. As a consequence, many cell surface proteins that need this anchor to attach to the plasma membrane are no longer available or only available in reduced numbers on the cell [3, 4]. Some of these proteins such as CD55 and CD59 are essential for the protection of red blood cells from complement mediated lysis. As a consequence, erythrocytes that lack CD55 and CD59 undergo intravascular hemolysis leading to anemia, hemoglobinuria, iron deficiency and fatigue. Scavenging of nitric oxide by free plasma hemoglobin results in endothelial and platelet dysfunction leading to the high risk of venous and arterial thrombosis associated with this disease. Additional symptoms related to nitric oxide depletion include abdominal pain, esophageal pain, chronic kidney disease and erectile dysfunction.

PNH is a rare condition and many practitioners (mostly those working outside of large hospital facilities) have yet to encounter a single patient with this disease. Although the discovery of somatic mutations in the PIG-A gene (which resides on the X chromosome) provided a very elegant explanation of how an acquired mutation in a single gene could lead to the disease phenotype [1], this does not explain how the mutant clone expands. It has been shown that (i) the mutation rate in PIG-A deficient cells is normal [5], (ii) PIG-A deficient cells are not more resistant to apoptosis than normal cells [6], (iii) the replication rate of mutated cells is normal [7] and PIG-A deficient cells do not have a proliferative advantage over normal cells [8].

Perhaps it was natural for investigators in the field to assume from the outset that there must be a selective fitness advantage of PIG-A mutated cells and consequently to investigate possible causes for some benefit that enables clonal expansion. It has been proposed that the selective advantage of mutated cells is extrinsic to them and due to an immune mediated attack on *normal* cells [9]. Some evidence in support of this hypothesis exists [10–13], but this hypothesis is unable to explain the following observations: (i) PIG-A is ubiquitously expressed in the body–why should the immune attack presumably against the GPI anchor be restricted to the *normal* HSC population? (ii) Immunosuppressive therapy does not lead to the elimination of the mutant cells and expansion of the normal HSC with return to normal hematopoiesis. (iii) A significant fraction of patients with PNH undergo ‘spontaneous’ extinction of the clone [14].

A second hypothesis has been proposed, which postulates that additional mutations in one or more genes that confer a fitness advantage to the PIG-A mutant cells may occur. Indeed, several case reports are available including two patients with a mutation in HMGA2 [15], one patient with a concomitant JAK2^V617F^ mutation [16], a mutant N-RAS [17] and more recently a patient with PNH and concomitant BCR-ABL in the same cell population was also reported [18]. However, these cases appear to be the exception and not the rule, and their description in our opinion requires specific explanations other than the one responsible for the general origin: clonal expansion and possible elimination of PNH phenotype clones. We have previously shown that given the normal mutation rate in PNH cells, it would be rare for a patient to have a second mutation in a PIG-A mutated stem cell that would provide the fitness advantage necessary for clonal expansion [19, 20]. Moreover, to date there is no evidence of a fitness advantage for the PIG-A mutated cells even though recent data suggests that some could have additional mutations (that are typically seen in myelodysplastic syndrome or leukemia) present [8, 21]. Finally, deep sequencing of patients with PNH clones reveals that a significant fraction do not have additional mutations present apart from that in PIG-A [21].

Given these observations, we proposed that perhaps clonal expansion in PNH is simply due to neutral drift, given i) that cells with GPI deficiency do not seem to have any evolutionary advantage over normal cells and ii) the limited data in support of any benefit with immunosuppressive therapy (in the absence of concomitant aplastic anemia) [22]. Interestingly, in some patients with PNH more than one clone is detected–one with complete deficiency of GPI anchored proteins (so called PNH III cells) and another with partial deficiency of GPI anchored proteins (PNH II cells) [3, 23] and this has been confirmed by sequencing [21] (for historical reasons, normal cells are referred to as PNH I cells). Clearly, these two mutant populations arise due to independent mutations in the PIG-A gene in different cells leading to these phenotypes.

In this work, we use evolutionary principles and stochastic dynamics modelling of the HSC pool to determine the incidence of the disease in populations, estimate clone size and average age at diagnosis, and also address the question of multiple PIG-A mutated clones in patients with this disease. These results strengthen and extend the ones found by Dingli et al. [22], who first tested this hypothesis, by the use of a Markov chain formulation rather than traditional simulations. We provide exact solutions for the probabilities in the state space and for the first time, an estimate of the rate of clonal expansion in this disease.

## Results

Taking the replication rate of HSC at 1/cell/year and the known normal mutation rate of cells ($5\times 10^{-7}$/replication) [5], numerically evolving the Markov chain gives us probabilities for all possible size combinations of the mutant clones. While some of the analyses given below were already performed by Dingli et al. in their initial work [22]–such as the estimations of disease prevalence and average clone size–the current approach allows for a more detailed and robust picture of the dynamics, as the probabilities of the state space are calculated exactly rather than inferred from simulations, allowing for a more accurate estimation of the rarest events such as the possibility of multiple separate clones in the HSC pool, the occurrence of PNH during childhood, and the spontaneous loss of a clone due to neutral drift. Furthermore, the expansion rate of the mutated clone is examined as well.

### Probability and incidence of clinical PNH with one or more clones

Depending on the total number of mutated stem cells in an individual we can assign different diagnoses: when at least 20% of the total stem cell pool has a PIG-A mutation the individual is defined as having *clinical* PNH. Cases where the PIG-A clone(s) consist of <20% of the cell population are considered to be *subclinical* (or latent) PNH [24].

**Fig 1:**
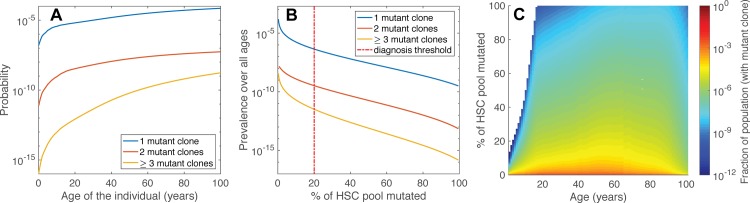
Dynamics of mutant cells and incidence of PNH derived from in silico studies. **A.** The probability of clinical PNH occurring in an individual between the ages of 1–100 years. Blue: patients with one active clone. Red: patients with 2 active clones. Yellow: patients where 3 or more active clones occurred. **B.** Expected incidence of clinical PNH in the US population, found by folding the Markov chain probabilities for ages 1–100 with population data from the 2010 US census. Same color codes as in **A**. **C.** The distribution of all individuals with a mutant clone in the 2010 US census population over the ages 1–100 and clone sizes 1%-100%.

The probability for an individual to develop clinical PNH increases with age according to the curves shown in Fig 1A, although this risk actually decreases briefly during the period of ontogenic growth due to the corresponding growth of the stem cell pool [25]. Indeed, the limited data available suggests that PNH is quite rare in children [26, 27] and generally occurs in the context of bone marrow failure. While classical hemolytic PNH represents about 10% of pediatric patients with a PIG-A mutant population, data from the International PNH registry suggests that perhaps half of adults with PNH have classical hemolytic disease [28].

We find that the probability of a patient having clinical PNH with two independent clones arising from the HSC pool is approximately $10^{3}$ times smaller than the same probability of diagnosis with a single clone (Fig 1A). Furthermore, we estimate that patients who have 3 or more distinct PNH clones contributing to hematopoiesis occur with a probability that is another 2 orders of magnitude lower (Fig 1A). This implies that approximately only 1 in 1000 cases of clinical PNH would host more than a single mutant clone that arose in the stem cell compartment. Note that these numbers result from a model dealing only with *stem cell* dynamics. Thus, this does not preclude the occurrence of mutations farther downstream among progenitor cells (which are present in larger numbers than HSC and also divide faster [19, 29]). Moreover, PIG-A mutations occurring in early progenitors will also remain contributing to hematopoiesis for years before any eventual wash-out [30, 31]. Thus, divergent PIG-A mutations found in mature cells are more likely to have originated at later stages of differentiation [19] than to originate in independent mutations occurring in the active stem cell population.

Using population age distribution data obtained in 2010 by the United States Census Bureau, we estimate the prevalence of clinical PNH (weighted sum of census data and clonal existence probabilities) for both mono- and multiclonal cases in the USA (Fig 1B and 1C). We calculate an expected prevalence of 1.76 cases per $10^{5}$ citizens for any diagnosis of clinical PNH (mono- or multiclonal), which is similar to what has been reported in a well-defined population by Hill et al. [32]. The expected number of patients with biclonal disease arising at the level of the HSC is determined at 1.29 per $10^{8}$ individuals. For the US population, this would amount to approximately 3000 patients with a single clone and 2 patients with biclonal disease, respectively. The number of individuals in the population with a subclinical (<20%) PIG-A mutated clone is estimated to be much higher, at 6.0 per $10^{4}$ for monoclonal and 1.9 per $10^{7}$ for biclonal cases, which amounts to respectively 184,495 and 60 individuals in the US.

### Arrival times of mutated clone and clinical PNH

**Fig 2:**
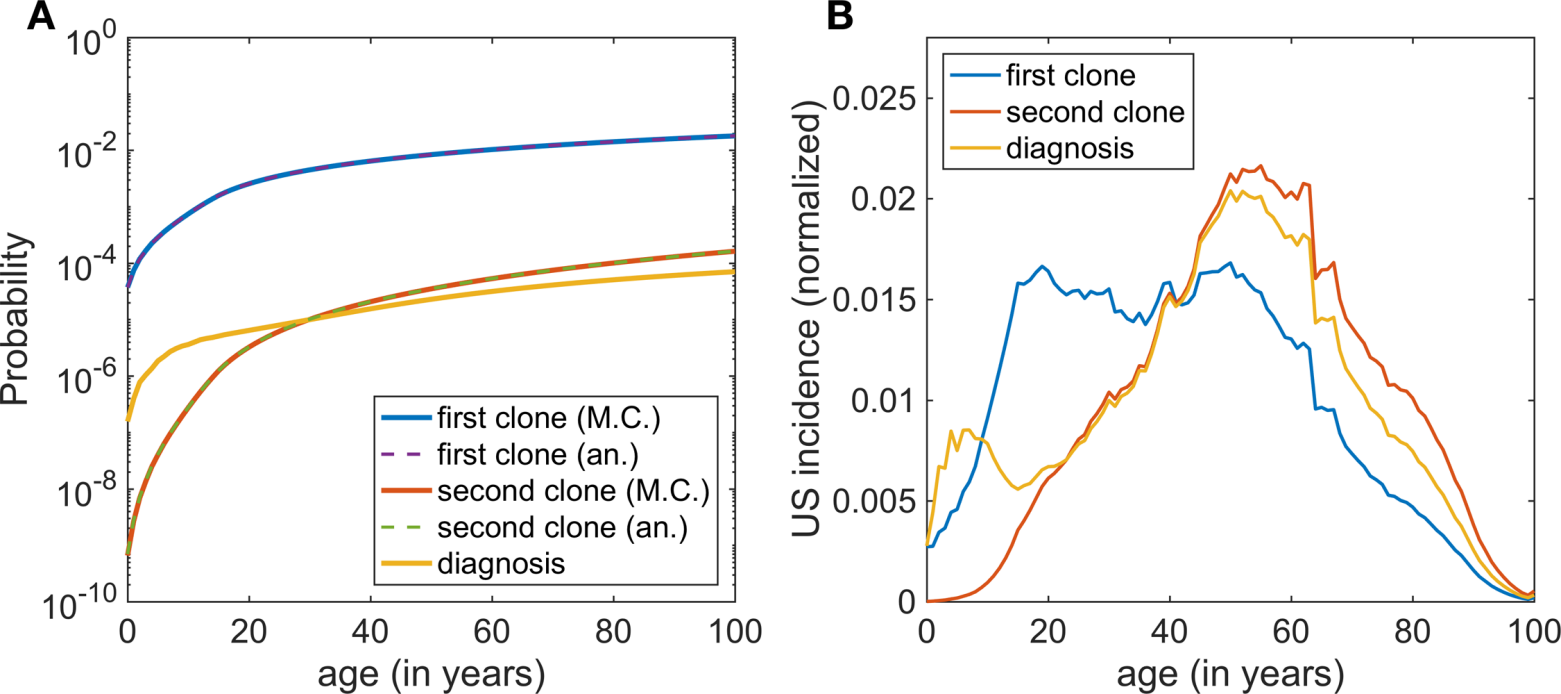
**A.** Likelihood of existence of clones over time. As a test of accuracy, the probabilities for the existence of the primary and secondary clones were also calculated analytically from a cumulative negative binomial distribution. Although the probability of harboring a clone is certainly non-negligible for most age groups, it is clear that the probability of diagnosis is many orders of magnitude smaller. **B.** The probability of obtaining a first or second clone in a given year as well as the probability of reaching the diagnosis threshold (20% of the HSC pool) folded with the 2010 US population distribution. The prevalence of every curve has been normalized to 1, so that these results may be interpreted as the age distribution of the clone and diagnosis arrival times. (M.C.: Markov Chain simulations; an.: Analytical calculations.)

The first mutated cell in the HSC pool can occur quite early in an individual’s life, as shown in Fig 2A. The probability of harboring a mutant cell in the stem cell population grows one order of magnitude from age 20 (~$2\times 10^{-3}$) to age 100 (~$2\times 10^{-2}$). Though these values may seem quite high, it is important to note that in the neutral drift hypothesis, the second line of defense against PNH is the significant low likelihood of clonal expansion, a fact that is illustrated well by comparing the probability of occurrence of a clone (which is quite common in healthy people [33]) with the probability of having clinical PNH. For example, in an individual of age 60, the probability of having acquired a mutant clone is $1\times 10^{-2}$, while the probability of having clinical PNH is $2\times 10^{-5}$, three orders of magnitude smaller. The average ages of clonal occurrence are projected at 41 and 54 years for mono- and biclonal (stem cell) cases respectively (Fig 2B). In general, it appears that, on average, most clones arrive only after adulthood is reached and the hematopoietic stem cell pool has reached its maximal size.

The average age at diagnosis–in our model we take this as the time at which the total number of mutated HSCs reaches 20%–is found to be 49 years, and is quite similar to what has been reported from the International PNH registry [28, 34]. Because some investigators define clinical PNH at a lower threshold, especially in the presence of aplastic anemia, we also calculated the average age when 10% of the HSC pool is composed of PIG-A mutant cells, and obtained a mean arrival time of 44 years.

### Clonal expansion under neutral drift acts like (frequency-dependent) Brownian motion

As mentioned above, the lack of a selective advantage makes it difficult for the mutated clone to expand, since at each replication event it is equally likely to decrease in size as it is to expand (if one neglects the low probability of mutation) [35–37]. Over time the size probability distribution widens, adding more emphasis on larger clones while smaller clones become less probable (since they are more likely to go extinct). Thus, in cases where two separate clones are simultaneously present, the first that occurred is likely to be larger and therefore less likely to resolve than the second.

From a mathematical perspective, this behavior can be ascribed to the fact that the all-normal state (absence of mutants) and all-mutant state (complete takeover by mutants—fixation) are (in the absence of mutations) absorbing states of the evolutionary dynamics. An important consequence of the all-normal absorbing state is that most clones which arise in a population go extinct before reaching a significant size. We find that approximately 83% of all clones that appeared in our in-silico population resolved, and in most of these cases clonal extinction would have occurred soon after the clone’s arrival, so that the individuals at stake would never have been diagnosed with PNH as their clone would have been very small. On the other hand, extrapolating these simulations to the entire hematopoietic tree clearly suggests that the massive cell turnover (with mutation) that occurs normally in hematopoiesis explains why finding a PIG-A mutant cell population with sensitive sequencing or flow cytometry in a healthy individual is not unexpected [33].

**Fig 3:**
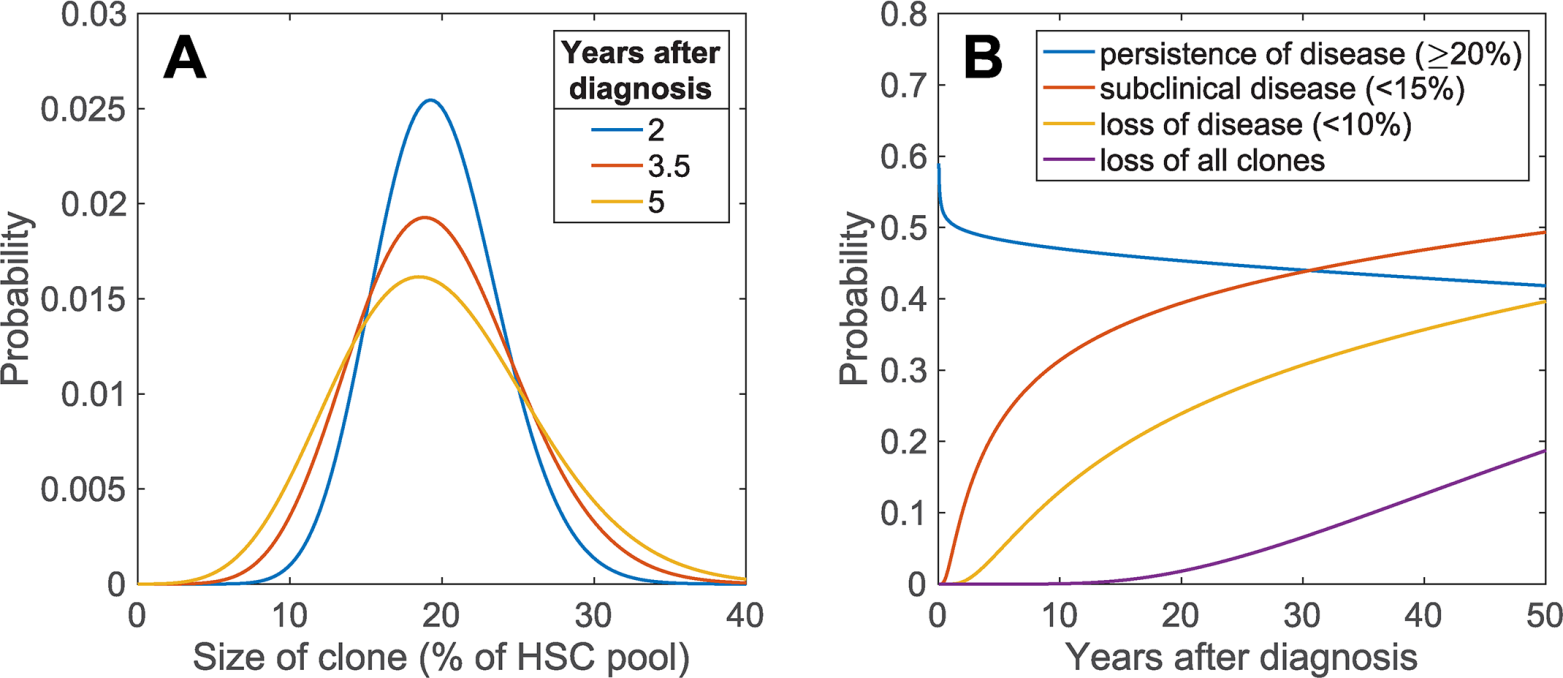
**A.** The size probability distribution for an established clone multiple years after diagnosis (20%). Being a one hump function, the distribution is asymmetric, stretching further to the right (larger sizes, see main text for details). **B.** Probabilities for an established clone to recede or vanish after diagnosis (20%) over time.

If a clone does manage to increase in size, the likelihood of spontaneous clonal extinction becomes less pronounced. In Fig 3A we show the probability distribution of clonal size measured at 3 different times after disease diagnosis. The variance of this distribution increases not only over time–as the clone has more time to expand or diminish–but also as the clone increases in size due to the frequency dependence of this “random walk”. In particular, the closer the clone size comes to comprising 50% of the SC pool, the larger this variance will be. One consequence of this behavior is that the distributions shown in Fig 3A are skewed to the right. Note, however, that despite the changing shape of the size distribution, the mean clone size (the average of this distribution) does not change over time. This result implies that in a cohort of diagnosed patients, we expect the average of their clone sizes to remain stable despite individual expansions or recessions.

### Average clone size and disease reduction

Using the census data, the average clone size $\langle m\rangle$ in individuals in the US population with at least one mutant HSC is estimated in our model to be at 3.4% of the total pool. However, the average clone size in those suffering from clinical PNH (m≥20%) is much larger, found to be 31.1%, or 19.6% if this threshold is instead taken at m≥10%. Of course, in individual patients, clone size can be quite high and be the predominant contributor to hematopoiesis.

Whenever the number of mutant stem cells reaches the threshold of clinical PNH, it is nevertheless possible for the disease to disappear due to the stochastic nature of the clonal dynamics. We calculated the probability that a recently diagnosed case of clinical PNH (assuming the SC pool is ≈20% mutated at diagnosis) becomes subclinical again. The result (Fig 3B) shows that over time it is more likely for the disease to recede than to persist, although it would take at least 2–10 years for significantly smaller clone sizes (<15% or <10%) to be reached. The probability of the clone becoming truly extinct only becomes realistic after 20–50 years, and in reality clinically detectable extinction will depend on the assay that is used to determine the presence or absence of the clone. It should be evident that more sensitive flow cytometry based assays will be able to detect the clone, even when the ‘old’ standard Ham’s test becomes negative.

Note that these results only represent the likelihood of disease reduction under neutral dynamics, and as such do not exclude the possibility of a more fit clone arising which can advantageously compete with the PIG-A clone and lead to disease loss [38].

### Validation with clinical data

We compared our predictions of average clone size and patient age at diagnosis with data from the International PNH Registry [28]. Our predicted mean clone size of 31.1% (standard deviation: 32.6) and mean age at diagnosis of 48.6 (standard deviation 52.8) seem to fit nicely with the registry data for patients with AA-PNH syndrome (categorized as also suffering from *aplastic anaemia*) which shows a mean clone size of 28.3% (standard deviation 32.8%) and 43.2% of patients diagnosed between 30 and 59 years of age. However, other categories presented much greater average clone sizes (though similar ages at diagnosis).

Our prediction of the number of patients with clinical PNH in the general US population is slightly lower than what Hill et al [36] reported. However, we note that our modeling takes into account the age structure of the population in the United States, whereas the patients observed by Hill et al. were from Great Britain, which may have a different age distribution. Furthermore, our prevalence estimates depend on a strict definition of clinical PNH (clone size >20%), whereas in clinical practice, the transition from subclinical to clinical disease may be less abrupt.

We also compared our findings on clonal expansion rate with measurements from Araten et al. [7], who reported a ≥5% size increase per year in 12 out of 36 patients. Most of the other patients experienced either a reduction or no change at all, though the authors did not specify these amounts quantitatively. The study found no significant expansion or reduction (≈0%) when calculating the mean over all patients, which nicely fits our neutral drift model. Our model projected the fraction of patients that would experience a ≥5% increase after 1 year to be between 5% and 10% depending on the size of the initial clone, which is lower than their observed 33%. This discrepancy could be due to several factors including the relatively small size of their patient cohort and the fact that our model does not include the contribution of progenitor compartments to the overall size of the PNH population. If the progenitor cell population is contributing significantly to hematopoiesis, then more fluctuations in clone size would be expected due to the shorter lifetime of these cells [30].

Our modeling predicts that after 10 years from diagnosis, the probability that the clone is small enough not to be associated with clinical PNH is upwards of 30% (Fig 3B). This is also comparable to what Hillmen et al [14] reported in their cohort of patients who survived for more than 10 years from diagnosis (12 out of 35 patients).

## Discussion

The appearance of mutations in HSC and their fate over time is an important clinical problem, since many diseases such as myelodysplastic syndromes and several leukemias (e.g. chronic myeloid leukemia, some subtypes of acute myeloid leukemia) arise due to mutations within the HSC. Landmark studies in PNH have shown that it is an acquired clonal HSC disorder [39] with very interesting dynamic properties, including an uncanny probability of spontaneous clonal extinction [14]. The mechanism of clonal expansion in PNH has been a source of great debate and several hypotheses have been proposed to explain it, such as a selective advantage of the mutant cells due to an immune attack on normal HSC (extrinsic advantage), or the presence of a second mutation that grants a fitness advantage (intrinsic advantage). Some evidence for either hypothesis exists, but both also suffer from deficiencies as described in the Introduction. In particular, immunosuppressive therapy does not return hematopoiesis to normal and there is no reduction in the size of the PIG-A mutant clone once the presumed selective advantage is eliminated. It is also difficult to see how a cell can acquire multiple mutations sequentially in the absence of genomic instability, which has not been observed in PNH [5]. We have proposed that the PIG-A mutant cells generally possess no fitness advantage (or disadvantage), and that clonal expansion is simply a consequence of neutral drift within the (small) active HSC pool that maintains hematopoiesis [22]. This hypothesis leads to the simplest of explanations of PNH, and our stochastic modeling suggests that this may be the case–at least in some patients–since we are able to predict the incidence and prevalence of the disease, average age at diagnosis, average clone size and the probability of clonal extinction purely from first principles with results quite similar to what has been reported in the literature. Although it is difficult to deliver conclusive proof of our hypothesis, the close parallel between our predictions and clinical reality provides considerable support for it.

It has been reported that in at least one patient, that PNH clonal extinction was concomitant with the appearance of a *new* population of cells that harbored mutations in genes such as STK36 that can potentially provide a fitness advantage to the non-PIG-A mutant cells [38]. Our model makes no assumptions about the possibility of more fit clones arising, but merely gives the likelihood of the disease disappearing entirely on its own through neutral drift. Moreover, while the ongoing dynamics within the normal hematopoietic stem cell group put this population at continued risk of accumulating new mutations–some of which could lead to a fitness advantage as proposed by Babushok et al [38]–the presence of such mutations by itself does not imply that the PNH clone resolved due to takeover of hematopoiesis by a new population with mutations that are often found in patients with myeloid disorders. As discussed elsewhere [40] the presence of such mutations by itself does not necessarily mean that the mutant cells with a normal PIG-A gene have a fitness advantage since the mutant gene (e.g. STK36) may or may not be a ‘driver’ mutation. This point is further highlighted by the fact that in the case reported by Bubashok et al [38], PNH clonal reduction occurred over the course of 12 years while our estimate for the general population under neutral drift is that on average, 8–10 years are required for the PNH clone to reach ~15%.

Neutral drift may come as a surprise for many in the field of hematology and oncology who are accustomed to associate malignant clonal expansions in cancer with some form of selective advantage. Nevertheless, it is not uncommon for mutations in populations to expand by neutral drift, as suggested by Kimura many years ago [41]. In fact, the recent cancer genomics data explosion suggests that most mutations found in tumours are examples of neutral drift and are labeled passenger mutations, since they provide no fitness advantage to the tumour [42]. Of course, mutations that increase the fitness of malignant clones clearly exist. Perhaps the main difference in PNH is that neutral drift could be the main mechanism of clonal expansion. Not only is it the simplest explanation, but it also provides a very elegant correlation of genotype with phenotype, a feat that is much more difficult to achieve in malignant tumours due to the complex mutational landscape that they harbor.

In conclusion, we present a stochastic model of HSC dynamics which studies mutations in the PIG-A gene that leads to the PNH phenotype. Our predictions based on an *in silico* model of Markovian stochastic dynamics enable us to determine from first principles the incidence of the disease in a population, the average clone size and the probability of clonal extinction, with results similar to what is observed in clinical practice. We also find that in a neutral drift model the probability of multiple PNH clones arising separately in the HSC pool is exceptionally small, a result which suggests that in clinical cases where differing clones are observed, all but one of the clones are likely to have emerged in later stages of differentiation. We propose that PNH is perhaps the first disease where neutral drift alone may be responsible for clonal expansion leading to a clinical problem.

## Methods

### Stochastic evolutionary model

The model describes a system of non-interacting HSC that undergo cell division and differentiation. The size $N_{SC}$ of this stem cell pool changes during ontogenic growth from about 20 cells at birth, to approximately 400 cells by adulthood [43], following the growth curve determined by Dingli et al [25, 44]. A stochastic evolution of the system is modelled in discrete-time steps, where at each time step a division and subsequent differentiation event are performed (so-called birth-death process). This is done by randomly selecting for replication a single cell from the stem cell pool, which generates two daughter cells. Since we consider only neutral dynamics, all cells possess the same fitness, meaning each cell (whether it is normal or PIG-A mutated) has the same probability of being selected. Following replication, a new cell is randomly selected from the total population (now of size $N_{SC}+1$) for differentiation, removing it from the stem cell pool. Thus $N_{SC}$ remains unchanged and this neutral evolution model can be considered a special case of the well-known Moran process [45]. When a normal cell replicates there is a probability μ that one of the daughter cells acquires a mutation, introducing a potential seed for the development of a mutated clone in the population. The cells in the active HSC compartment, replicate slowly, at a rate of approximately once per year [46]. The conversion between number of cell replications and biological time is done in the following way: When the number of replication events equals the cell population size, then one year has passed. Thus, in adulthood, if ≈400 replications within the HSC pool occurred, a year would have passed. The increase of $N_{SC}$ during ontogenic growth follows an predetermined growth curve derived before [25, 44] and is implemented by including additional divisions without successive differentiations at fixed times (2 week intervals) to match the expected growth rate. Thus, while the Moran-like dynamics are respected for all division-differentiation events, they are periodically interrupted during this phase to model the increase of the population.

While this model of neutral dynamics has already been introduced and studied by Dingli et al. [22], the current approach (described below) allows for a more robust probing of the system from which new insights can be obtained.

### Markov chain formulation

The discrete time-evolution of this system is well described by a Markov chain in which the state space is represented by the number of mutants present in the population. To predict the stochastic evolution we numerically evolve the master equation

$$P_{m}\left[x+1\right]=\underset{k}{\sum }p_{m,k}\;\times \;P_{k}\left[x\right]$$

where $P_{m}\left[x\right]$ is the probability of finding the HSC cell population in state m (with *m* being the number of mutated cells in the population) at time step x, and $p_{m,k}$ is the probability to go from state k to state m in a single time step. Starting from a mutant free population leads to the initial conditions $P_{0}\left[0\right]\;=\;1$ (the probability of 0 mutants existing at time *t* = 0 is 1) and $P_{m>0}\left[0\right]\;=\;0$ (the probability of more than 0 mutants existing at time *t* = 0 is 0). The transition probabilities are found by considering all changes that may occur when performing a division and differentiation event. For example, an event in which a normal cell divides without acquiring a mutation and a normal cell differentiates results in a transition into the same state (one normal cell is added and one is removed), and occurs with a probability $\left(1-\frac{m}{N_{SC}}\right)\left(1-\mu \right)\left(1-\frac{m}{N_{SC}+1}\right)$. The total probability of staying in the same state (i.e. the transition m→m) is found by summing this term with all other events that result in a same state transition; in particular, if a mutant cell is selected in both division and differentiation steps–probability $\frac{m}{N_{SC}}\frac{m+1}{N_{SC}+1}$, and if a normal cell divides but acquires a mutation and a mutant cell differentiates–probability $\left(1-\frac{m}{N_{SC}}\right)\mu \frac{m+1}{N_{SC}+1}$. In a similar manner we can obtain all nonzero elements of the transition matrix to obtain:

$$\{\begin{array}{c} p_{m,m-1}=\frac{m-1}{N_{SC}}\left(1-\frac{m}{N_{SC}+1}\right)+\left(1-\frac{m-1}{N_{SC}}\right)\mu \left(1-\frac{m}{N_{SC}+1}\right)\\ p_{m,m}=\frac{m}{N_{SC}}\frac{m+1}{N_{SC}+1}+\left(1-\frac{m}{N_{SC}}\right)\mu \frac{m+1}{N_{SC}+1}+\left(1-\frac{m}{N_{SC}}\right)\left(1-\mu \right)\left(1-\frac{m}{N_{SC}+1}\right)\\ p_{m,m+1}=\left(1-\frac{m+1}{N_{SC}}\right)\left(1-\mu \right)\frac{m+1}{N_{SC}+1} \end{array}$$

Note that for the “division-only” events occurring sporadically during ontogenic growth a simpler transition matrix is used in which no differentiation takes place (see S1 Text). It is clear that only transitions between “nearest-neighbours” in the state space are possible, so that $p_{m,k}\;=\;0$ if $\left|m-k\right|>1$.

An interesting property of this system can be seen from that fact that if we neglect the possibility of a new clone arising (that is, neglecting the possibility of mutation so that μ = 0), the transition probabilities to move up or down from a state m in a single time step are identical:

$$p_{m+1,m}=p_{m-1,m}=\frac{N_{SC}m-m^{2}}{N_{SC}\left(N_{SC}+1\right)}$$

This symmetry implies the system will perform a random walk reminiscent of Brownian motion, the main difference being that the probability to move away (up or down) from a current state is not independent of m, instead adhering to the quadratic function given above, with a maximum value at $m\;=\;N_{SC}/2$, reflecting the frequency dependence of the evolutionary dynamics. This means that the closer the mutant population gets to this maximum, the more “volatile” it becomes.

It is also worth noting that while the above transition matrix holds–as mentioned earlier–only in the absence of fitness differences between cell types, it can easily be extended to cases where one type is more likely to divide or differentiate. The probability of a cell selected for division (or differentiation) being a mutant then not only depends on the size m of the mutant clone as $m/N_{HSC}$, but also on the clone’s relative fitness $r_{PIGA}$ [47]

$$P\left\{dividing\;cell\in mutants\right\}=\frac{r_{PIGA}m}{r_{PIGA}m+r_{healthy}\left(N_{HSC}-m\right)}$$

Where $r_{healthy}$ is the fitness factor of the non-mutated HSC. It is also immediately clear that taking $r_{PIGA}\;=\;r_{healthy}$ reduces the expression to the selection-free case treated in this work. The entire set of transition probabilities in the presence of selection are given in the appendix.

### Observing multiple clones

Evolving the basic Markov chain described above provides no method for tracking the evolution of multiple clones that arise from separate mutational events, as only a single PIG-A mutant population is considered. Thus, in order to distinguish clonally unrelated subpopulations, we extend the model by expanding the state space to account for multiple mutations. In the following, we take advantage of previous estimates which show that one can safely ignore two mutations in the same stem cell [19, 20] to limit our state space to account for a maximum of two different clones. This considerably simplifies the associated computations, as the state space scales as ${\sim N}^{i}$, with N being the cell population size and i the maximum number of independent clones. As a result, the state space is divided into three separate *histories*, as shown in Fig 1, corresponding to states with different pasts.

**Fig 4:**
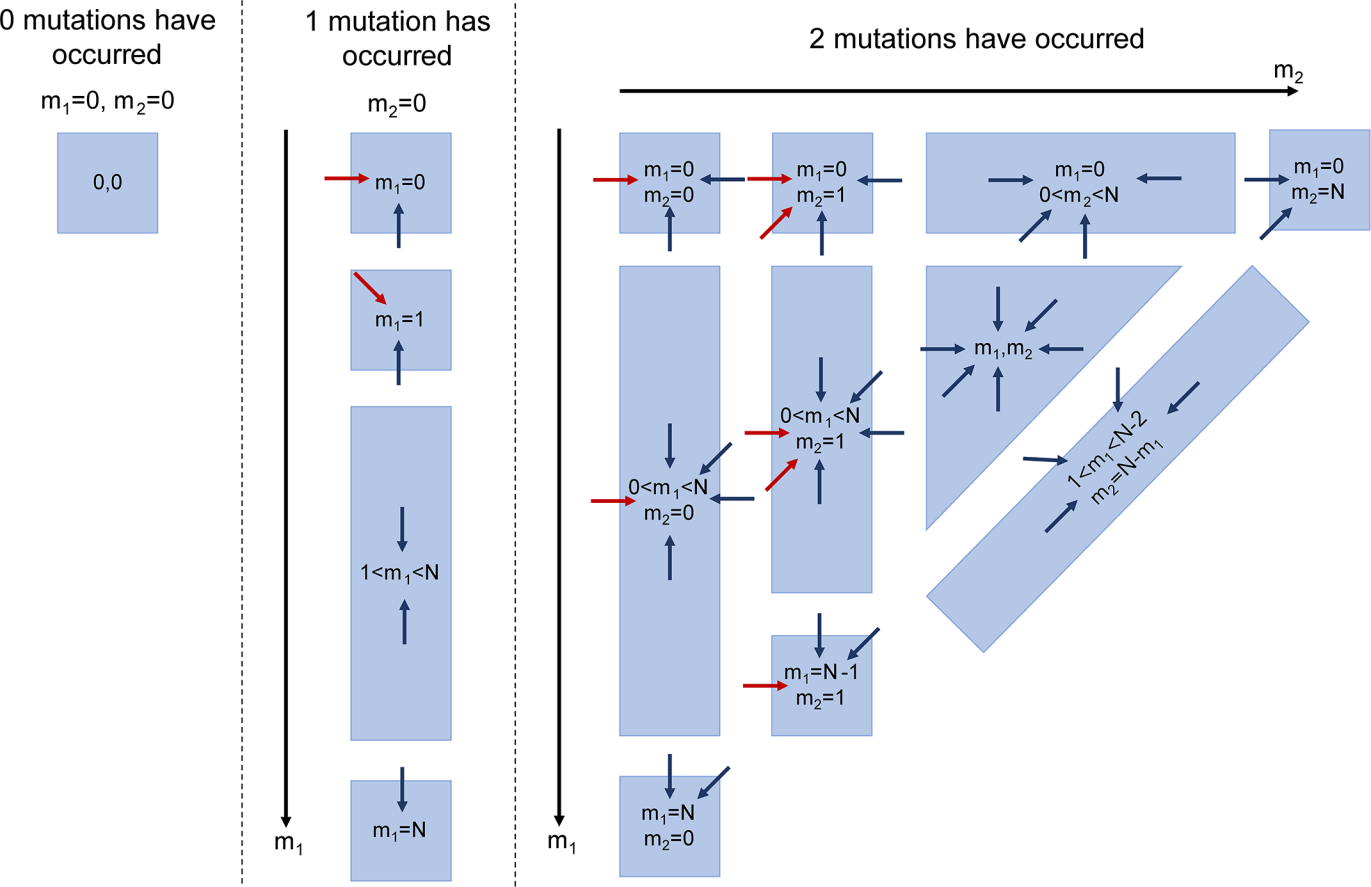
The state space and allowed transitions. Each history describes a possible evolution where either 0 (left), 1 (middle) or 2 (right) mutations were acquired by healthy cells. Whenever a mutation occurs the system jumps to the next panel on the right (to the next history), until the final history is reached where mutations are no longer allowed. The dark blue arrows represent incoming transitions from nearest neighbor states in the same history, while the red arrows represent transitions from states in the previous history. Note that every state also has a transition onto itself, which has been left out for readability.

For example, an evolutionary history in which a mutation occurred but the resulting clone eventually died should correspond to a different state than one where no mutation occurred in the first place. Thus, the master equation is now altered to include transitions to different histories:

$$P_{m_{1},m_{2}}^{i}\left[x+1\right]=\underset{m_{1}^{'},m_{2}^{'},j}{\sum }p_{m_{1},m_{2},m_{1}^{'},m_{2}^{'}}^{i,j}\times \;P_{m_{1}^{'},m_{2}^{'}}^{j}\left[x\right]$$

where any state is now characterized by the clone sizes $m_{1}$ and $m_{2}$, and the appropriate history i. The tensor elements $p_{m_{1},m_{2},m_{1}^{'},m_{2}^{'}}^{i,j}$ represent transitions from state $\left(m_{1}^{'},m_{2}^{'}\right)$ to ${(m}_{1},m_{2})\;$and history j to i, and are found in an identical manner as before, though now more transitions are possible (Fig 4).

### Average clone size calculation

The probabilities obtained by evolving the master equation also allow us to calculate the average clone size $\langle m\rangle$ in individuals of a given population whose clone size is within a chosen range. For example, in “clinical PNH” patients (defined as having a clone which involves at least 20% of the HSC pool) we are only interested in individuals whose number of PIG-A mutated cells ranges between 20% and 100%. The expression to evaluate is then:

$$m=\frac{\sum_{y=1}^{100}\sum_{m=m_{0}}^{N_{SC}}m\;w_{y,m}q_{y}}{\sum_{y=1}^{100}\sum_{m=m_{0}}^{N_{SC}}w_{y,m}q_{y}},$$

where $w_{y,m}$ is the probability of an individual of age y having a clone of size $m\;(w_{y,m}\;=\;\sum_{i,n}P_{m,n}^{i}\left[y\right]$ which results from evolving the Markov chain), and $q_{y}$ is the fraction of individuals of age y in the population based on the 2010 US census [United States Census Bureau. Single Years of Age and Sex: 2010. https://www.census.gov. Accessed 13 June 2017.], where we take individuals up to 100 years of age. The terms in the numerator form the average when all possible sizes $\left\{0,\ldots ,N_{SC}\right\}$ are considered, while the denominator normalizes the result if we are interested in a particular range, e.g. $\left\{m_{0}\to N_{sc}\right\}$ (it is easy to see that when $m_{0}\;=\;0$ the denominator becomes 1).

## Supporting information

S1 Text Supporting methods. (DOCX)
