# Supplementary material for: Evolutionary dynamics of paroxysmal nocturnal hemoglobinuria
Source: PLoS Comput Biol. 2018 Jun 18;14(6):e1006133. doi: 10.1371/journal.pcbi.1006133 (PMC6023248; doi:10.1371/journal.pcbi.1006133)
Supplement: S1 Text — (DOCX) [file pcbi.1006133.s001.docx]

# Supporting information

**Introducing fitness**

The transition elements $p_{m,n}$ for the Markov chain

$$P_{m}\left[ x+1 \right]=\sum_{k} p_{m,k} \times P_{k}\left[ x \right]$$

can be extended to incorporate fitness differences between cell types. To do this one must introduce the fitness parameters $r_{PIGA, div}$, $r_{PIGA,diff}$, $r_{healthy,div}$ and $r_{healthy,diff}$, which represent the mutated and normal cell’s likelihood of being selected for division and differentiation respectively. The actual selection probability is determined by the ratio of these parameters, so if for example $r_{PIGA}=2r_{healthy}$, a mutant cell is twice as likely to divide as a healthy cell.

$$\left\{ \begin{aligned} p_{m,m-1}=&\frac{r_{PIGA,div}\cdot\left( m-1 \right)}{\mathcal{N}_{div}\left\{ m-1,N \right\}}\times\frac{r_{healthy,diff}\cdot\left( N_{HSC}+1-m \right)}{\mathcal{N}_{diff}\left\{ m,N+1 \right\}} \\ &+\frac{r_{healthy,div}\cdot\left( N_{HSC}-m+1 \right)}{\mathcal{N}_{div}\left\{ m-1,N \right\}}\mu\times\frac{r_{healthy,diff}\cdot\left( N_{HSC}+1-m \right)}{\mathcal{N}_{diff}\left\{ m,N+1 \right\}} \\ p_{m,m}=&\frac{r_{PIGA,div}\cdot m}{\mathcal{N}_{div}\left\{ m,N \right\}}\times\frac{r_{PIGA,diff}\cdot\left( m+1 \right)}{\mathcal{N}_{diff}\left\{ m+1,N+1 \right\}} \\ &+\frac{r_{healthy,div}\cdot\left( N_{SC}-m \right)}{\mathcal{N}_{div}\left\{ m,N \right\}}\mu\times\frac{r_{PIGA,diff}\cdot\left( m+1 \right)}{\mathcal{N}_{diff}\left\{ m+1,N+1 \right\}} \\ &+\frac{r_{healthy,div}\cdot\left( N_{SC}-m \right)}{\mathcal{N}_{div}\left\{ m,N \right\}}\left( 1-\mu\right)\times\frac{r_{healthy,diff}\cdot\left( N_{HSC}+1-m \right)}{\mathcal{N}_{diff}\left\{ m,N+1 \right\}} \\ p_{m,m+1}=&\frac{r_{healthy,div}\cdot\left( N_{SC}-m-1 \right)}{\mathcal{N}_{div}\left\{ m+1,N \right\}}\left( 1-\mu\right)\times\frac{r_{PIGA,diff}\cdot\left( m+1 \right)}{\mathcal{N}_{diff}\left\{ m+1,N+1 \right\}} \end{aligned} \right.$$

Here the denominators are given by the shorthand (where $\alpha$ stands for $div$ or $diff$):

$$\mathcal{N}_{\alpha}\left\{ m,N \right\}=r_{PIGA,\alpha}\cdot m+r_{healthy,\alpha}\cdot\left( N_{HSC}-m \right)$$

One can easily check that if $r_{PIGA}=r_{healthy}$ these transition probabilities reduce to the ones given in the main text.
